# Acute kidney injury secondary to urinary tract infection in kidney transplant recipients

**DOI:** 10.1038/s41598-022-15035-7

**Published:** 2022-06-27

**Authors:** Tomasz Królicki, Klaudia Bardowska, Tobiasz Kudla, Anna Królicka, Krzysztof Letachowicz, Oktawia Mazanowska, Wojciech Krajewski, Paweł Poznański, Magdalena Krajewska, Dorota Kamińska

**Affiliations:** 1 Department of Anesthesiology and Intensive Care, Opole University Hospital, Opole, 45-401 Poland; 2 Department of Nephrology and Transplantation Medicine, Wroclaw Medical University, Wrocław, 50-556 Poland; 3 Department of Cardiology, American Heart of Poland, Kedzierzyn-Koźle, 47-200 Poland; 4 Department of Minimally Invasive and Robotic Urology, Wrocław Medical University, Wrocław, 50-556 Poland

**Keywords:** Kidney diseases, Renal replacement therapy, Acute kidney injury, Chronic kidney disease, Urinary tract infection

## Abstract

Acute kidney injury (AKI) in kidney transplant recipients (KTRs) is a common, yet poorly investigated, complication of urinary tract infections (UTI) and urosepsis. A retrospective comparative analysis was performed, recruiting 101 KTRs with urosepsis, 100 KTRs with UTI, and 100 KTRs without history of UTI or sepsis. The incidences of AKI in the urosepsis and UTI groups were 75.2% and 41%, respectively. The urosepsis group has also presented with a significantly higher prevalence of AKI stage 2 and 3 than the UTI group. The rates of recovery from AKI stages 1, 2 and 3, were 75,6%, 55% and 26.1%, respectively. Factors independently associated with renal recovery from AKI were: AKI severity grade (AKI stage 2 with OR = 0.25 and AKI stage 3 with OR = 0.1), transfusion of red blood cells (RBC) (OR = 0.22), and the use of steroid bolus in the acute phase of treatment (OR = 4). The septic status (urosepsis vs UTI) did not influence the rates of renal recovery from AKI after adjustment for the remaining variables. The dominant cause of RBC transfusions in the whole population was upper GI-bleeding. In multivariable analyses, the occurrence of AKI was also independently associated with a greater decline of eGFR at 1-year post-discharge and with a greater risk of graft loss. In KTRs with both urosepsis and UTI, the occurrence of AKI portends poor transplantation outcomes. The local transfusion policy, modulation of immunosuppression and stress ulcer prophylaxis (which is not routinely administered in KTRs) in the acute setting may be modifiable factors that significantly impact long-term transplantation outcomes.

## Introduction

Acute kidney injury (AKI) is a frequent complication of infections in the general inpatient population, with prevalence ranging up to 50% of cases in Intensive Care Units (ICUs), depending on the profile of the department^1,2^. The occurrence of AKI is significantly associated with in-hospital mortality, especially in ICUs, but also with a decline of long-term renal function in both native and transplanted kidneys^3–6^. The most common cause of AKI in kidney transplant recipients (KTRs) is urinary sepsis (US) followed by other urinary tract infections (UTI)^4^. For this study, the term: “UTI-related acute kidney injury” (UTI-AKI) has been coined. The urinary tract remains the most common infection site in this patient group, presumably due to the anatomic characteristics of transplanted kidneys^7–9^. These characteristic features include a short ureter, lacking gravity barrier for urinary reflux, and insufficient anti-reflux properties of vesicourethral anastomosis. As the post-transplantation occurrence of UTI is a known factor that impairs renal function, no studies have analyzed the prevalence of AKI in KTRs with UTI or urosepsis as well as their parallel impact on the allograft function^10–14^. Due to the paucity of clinical papers available at this point (data search performed in May 2021), the real renal burden of septic- and UTI-related AKI in KTRs is unknown. Except for anatomic characteristics of kidney allograft in KTRs, there are plenty of other specific factors contributing to the development of UTI and AKI. These include residual foreign bodies in the urinary tract such as ureteric stents or Foley catheter, urinary obstruction (due to prostatic hyperplasia, pelvic organ prolapse or congenital anomalies), malnutrition, urolithiasis, low urine output and many others^9,15,16^. Additionally, in KTRs the continuous immunosuppressant treatment also impairs the immune response to any ongoing inflammatory processes in the urinary tract, at the cost of optimal kidney allograft function. As infections remain the most frequent cause of death in KTRs, the intensity of immunosuppression must be always balanced with infection risk, for which statistical models have already been developed^6,17^. This study aimed to elucidate the incidence and identify the modifiable risk factors of UTI-related AKI in KTRs, with a focus on the impact of implemented treatment on long-term transplantation outcomes.

## Materials

### Study design

The study was designed as a retrospective observational study. The study group comprised of 301 KTRs including 101 KTRs hospitalized for urinary sepsis, 100 KTRs hospitalized for UTI, and 100 healthy KTRs serving as a control group. The follow-up time was set for 12 months, including 4 routine visits to the outpatient clinic. The permission for the use of retrospective data was obtained from appropriate authorities.

### Study groups

The study aimed to recruit all primary cases of urosepsis and about 100 KTR with non-septic UTI and 100 KTRs without history of prior UTI or US. For this purpose, hospital records for the years 2014 to 2019 were screened for cases of urinary sepsis in KTRs (using appropriate ICD-10 coding) identifying 148 potential cases. From this group, 101 cases were included in the urosepsis group, using the following inclusion and exclusion criteria:

Inclusion criteria:

- Symptomatic and laboratory-confirmed UTI
- Documented increase of SOFA (Sequential Organ Failure Assessment)—score of 2 points or more

Exclusion criteria:

- Allograft dysfunction caused by an acute disease other than UTI (for example, myocardial infarction, contrast agents, acute intraabdominal process, stroke, acute graft rejection, etc.) at the time of presentation
- Occurrence of another episode of urosepsis within 2 years before the presentation (to exclude the impact of another urosepsis on transplantation outcomes)
- A diagnosis of any disease with a life expectancy lower than 2 years

**Figure 1:**
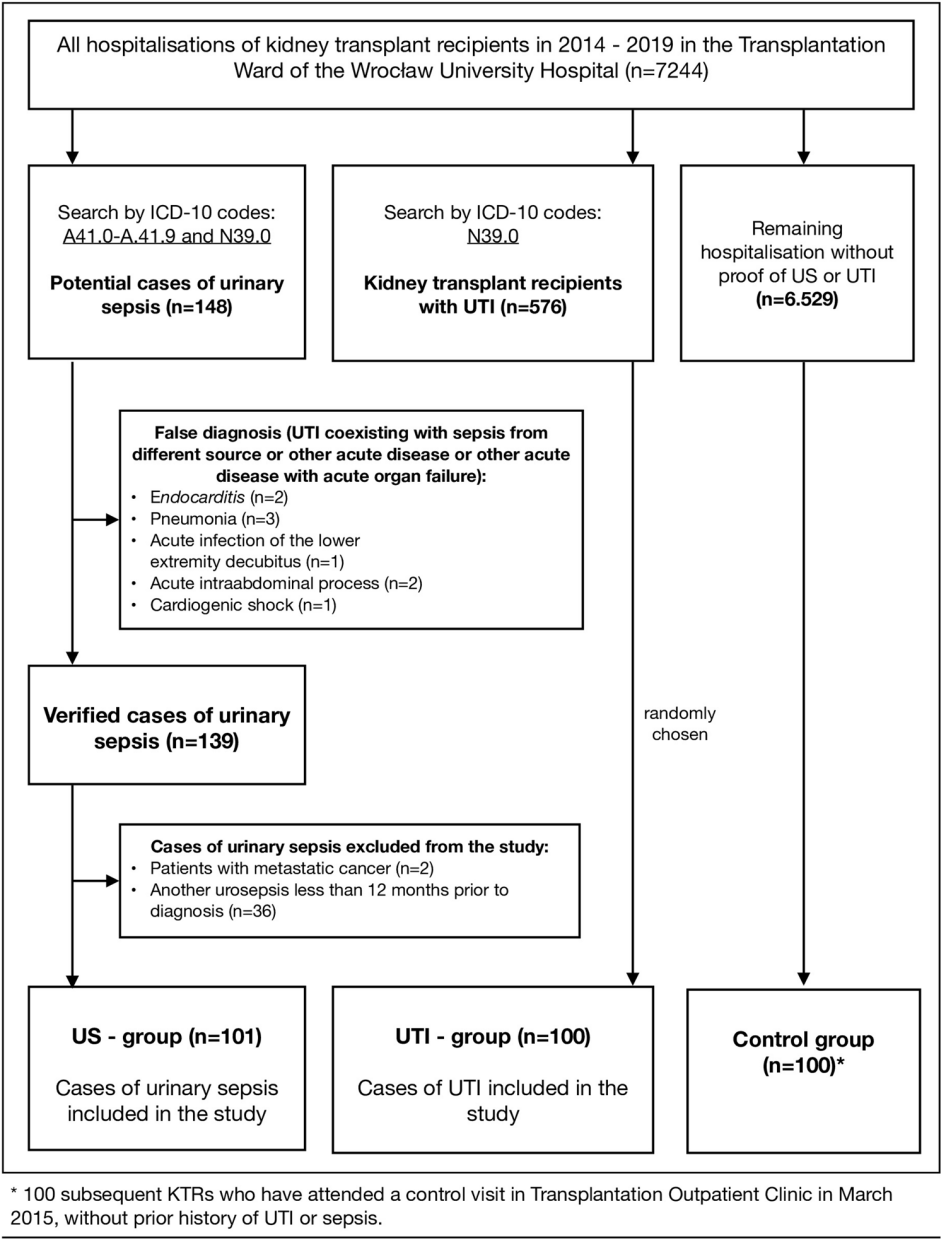
The selection process of patients into the study group.

The inclusion criteria were based on the European Association of Urology guidelines on Urinary Tract Infections^18^ and The Third International Consensus Definitions for Sepsis and Septic Shock^19^. The second group named the UTI group, comprised of 100 random KTRs hospitalized for non-septic UTI within the same timespan. The patients were selected randomly out of 576 KTRs hospitalized for UTI, using MS Excel coomands (“RAND” and “INDEX” functions). The third group which consisted of 100 healthy KTRs (with no history of UTI or sepsis) who attended a routine visit in March 2015, were used as a control group (CG). The selection process of patients to all 3 study groups is presented on the flowchart in Fig. 1.

### Methods

The clinical data regarding hospitalization was collected. The incidence of AKI was assessed using the KDIGO 2012 criteria^20^. A one-year follow-up was applied to all the study subjects using the records from the local outpatient transplantation unit. During the follow-up period, the incidence of graft loss and acute rejection, as well as allograft function after 1, 3, 6 and 12 months were recorded. Recovery from AKI was defined as a difference between creatinine levels at 1-month post-discharge and at baseline equal to or less than 0.3 mg/dL. Graft loss was defined as the return to chronic dialysis at any time of the follow-up period. Additionally, kidney function at 3 and 12-months before inclusion to the study was registered. eGFR values were calculated using the MDRD-4 formula. Baseline kidney graft function was defined as either last known eGFR measurement or a mean from two last eGFR values.

### The choice of formula for eGFR estimation

#### Literature review

There are several formulas destined for calculation of estimated effective Glomerular Filtration Rate (eGFR). The most popular ones are MDRD-4 and CKD-EPI, which rely on patients age, sex, ethnicity and serum creatinine concentration. Other formulas utilising patients weight or serum concentrations of other biomarkers as cystatine C have not been widely accepted as golden standard, despite being significantly more precise than the ones based on creatinine levels. Our choice of MDRD-4 over CKD-EPI formula for estimation of eGFR in this study has been motivated by previous literature search. Both formulas have been validated in the populations of KTRs^21,22^. The CKD-EPI has been shown to be the most accurate formula in general CKD population, however in KTRs it is the MDRD-4 that shows the lowest bias in comparison to a reference method^23^. In addition, the CKD-EPI formula seems to overestimate real eGFR values especially in patients with eGFR above $>60\;{\text {ml/min}}/1.73\;{{\text {m}}^{2}}$^23^.

#### Comparison of the calculated eGFR values with MDRD-4 and CKD-EPI formulas

We have performed a Bland-Altman analysis comparing eGFR values estimated by MDRD-4 and CKD-EPI equations. The eGFR calculated by both formulas have shown a wide range of agreement ranging from 0 up to 60–$70\;{\text {ml/min}}/1.73\;{{\text {m}}^{2}}$ of eGFR. The mean difference between eGFR calculated from CKD-EPI and MDRD-4 equaled 3.4 ± $3.1\;{\text {ml/min}}/1.73\;{{\text {m}}^{2}}$, which is concordant with experiences from large studies. Adequate Tukey plots from this analyzis were provided of Figs. S1–S6 in the Supplement to this article. Therefore, as 67% of the study patients at baseline have presented eGFR (MDRD-4) below $<60\;{\text {ml/min}}/1.73\;{{\text {m}}^{2}}$ we have decided that MDRD-4 formula seems to be best suited for the investigated patient population.

### Statistical analyzis

Binominal and discrete data were presented as total counts and percentages. The differences between these variables were tested using the Fisher exact test and Chi-square test. The study used the Shapiro-Wilk test for continuous variables to assess the assumption of normality and presented the results as either mean standard deviation (SD) or median and interquartile range (IQR), as appropriate. The significance of differences between the two groups was tested using the independent Student’s t-test for normally distributed variables and the Mann–Whitney U-test for skewed variables. For multiple comparisons, univariable ANOVA and Kruskal-Wallis ANOVA were implemented. For comparison of dependent variables, a dependent t-test and Wilcoxon test were used as appropriate. The risk factors of AKI were identified using a multivariable logistic regression model. To identify variables associated with outcomes, a multivariable linear regression, logistic regression and Cox hazards regression models were implemented. Some models were manually adjusted for variables which would not normally be included in the model (due to lacking statistical significance—aproppriate notes are included in Table captions if done so). The occurrence of events in time was presented using Kaplan Meier survival curves and differences between them were tested using the log-rank test. A two-tailed p-value of $<0.05$ was set to be statistically significant. All analyses were conducted using Statistica software version 13.2 (StatSoft inc., Tulsa, Oklahoma).

### Ethics approval

The study was approved by the local ethics committee of Wroclaw Medical University (approval number KB-775/2018), and the need for obtaining informed consent has been waived due to the retrospective character of the study. The study was performed in compliance with the Declaration of Helsinki.

### Consent for publication

Consent for retrospective use and publication of clinical data was obtained from the local data administrator (Wrocław Medical University, Department of Nephrology and Transplantation Medicine).

### STROBE statement

The manuscript complies with the STROBE checklist for reporting of observational studies. Filled checklist was attached to the manuscript.

## Results

The baseline characteristics of the urosepsis, UTI and control group are presented in Table 1. Patients with urosepsis were slightly older than their counterparts from the UTI and control group. The baseline serum creatinine concentrations did not differ across all three groups, neither did the calculated eGFR values (urosepsis: 45.5 ± 17.59 vs UTI: 48.3 ± 13.82 vs CG: 50.8 ± 14.54, p = 0.0647 for trend). Patients with urosepsis have also presented significantly longer hospital stay as compared to UTI patients [14 days (10–20) vs 8 days (6–13), $p<0.0001$] and a significantly more frequent history of recurrent UTI before hospitalization (28.7% vs 9%, p = 0.0002)—as defined by EAU^18^. The in-hospital mortality was observed only in the urosepsis group and reached 8%. All-cause mortality at 1 year post-discharge in all 3 groups equaled 1%. The 1-year death-censored graft loss (DCGL) was significantly higher in the urosepsis group as compared to both UTI and control group (17.4% vs 10.1% vs 5%, p = 0.0183). After 1 year of follow-up, the urosepsis group also exhibited the lowest eGFR, followed by the UTI group, and the highest eGFR was observed in the control group (US: 37.1 ± 16.54 vs UTI: 44.8 ± 17.09 vs CG: 50.0 ± 14.84, p $<0.0001$ as presented in Table 1. The incidence of acute rejection did not differ significantly across the groups at 1 year. The study also reports high urosepsis recurrence rates (39.1%) and a significantly higher incidence of subsequent UTI episodes in the urosepsis group (US: 47.5% vs UTI: 22%, p $<0.0001$) during the follow-up period. The incidence of organ injury and failure in both the urosepsis and UTI groups is presented in Table 2. The overall incidence of these events was higher in the urosepsis group. The incidence of AKI in the urosepsis and UTI groups equaled 75.2% and 41% respectively. The urosepsis group presented with a significantly higher proportion of patients with stage 2 AKI (15.8% vs 4%, p = 0.0102) and stage 3 AKI (20.8% vs 2%, p $<0.0001$). Renal replacement therapy was initiated in 17 cases in the urosepsis group and only in one case in the UTI group. Five of these subjects (admitted to ICU) were treated with continuous techniques (continuous veno-venous hemofiltration) and in the remaining patients, intermittent hemodialysis was applied. Despite not fulfilling the criteria of urosepsis, some patients in the UTI group have also presented with a degree of multiorgan dysfunction. These cases were not classified as urinary sepsis, as they were deemed to be not UTI-associated or have occurred late and were attributed to secondary complications related to hospitalization (pneumonia, decubitus formation). It is also worth mentioning that pulmonary congestion (in clinical examination) on admission to Transplantation Unit was significantly more frequent in the urosepsis group as compared to their UTI counterparts (20.8% vs 9%, p = 0.0283). No differences regarding the presence of peripheral edema, urinary obstruction and the need for urinary tract instrumentation were noticed. The urosepsis group has, however, presented a significantly more frequent need for packed red blood cell (PRBC) transfusions (14.9% vs 3%, p = 0.0070). Trends in the management of immunosuppressive regimens also differed significantly between the urosepsis and UTI groups (lower section of Table 2).

**Table 1:** Baseline characteristics of the study groups and endpoints occurring during follow-up.

	Urosepsis (n = 101)	UTI (n = 100)	CG (n = 100)	p-value
**General characteristics**				
Age (years)	58 (44–66)	56 (44–66)	56 (43–64)	0.0193
Sex (males/females)	47/54	42/58	68/32	0.3068
BMI (kg/m$^{2}$)	25.6 (21.4–29.7)	24.6 (22.5–27.9)	25.5 (22.8–29.1)	0.0555
Length of stay (LOS) (days)	14 (10–20)	8 (6–13)	–	$<0.0001$
Charlson Comorbidity Index	5 (3–6)	4 (3–5)	4 (3–5)	0.0600
History of reccurent UTI	29 (28.7%)	9 (9%)	–	0.0002
Time from KTx (months) to admission	39.5 (4.7–130)	49.1 (13.1–140)	47 (12–121)	0.5254
Baseline creatinine (mg/dL)	1.56 0.57	1.43 0.43	1.49 0.51	0.1271
Baseline eGFR (ml/min/$1.73\;{m}{^{2}}$)	45.5 17.59	48.3 13.82	50.8 14.54	0.0647
**Endpoints**				
Primary endpoints				
In-hospital mortality	8 (7.9%)	0 (0%)	–	0.0120
1-year eGFR [ml/min/$1.73\;{m}{^{2}}$]	37.1 ± 16.54	44.8 ± 17.09	50.0 ± 14.84	0.0001
1-year death censored graft loss^a^	16 (17.4%)	10 (10.1%)	5 (5%)	0.0183
1-year acute rejection (AGR)^a^	3 (3.2%)	3 (3%)	1 (1%)	0.4965
**Secondary endpoints**				
Recurrent UTI^a^	15 (16.3%)	1 (1%)	–	0.0008
1-year mortality post discharge	1 (1%)	1 (1%)	1 (1%)	0.9999
Another urosepsis^a^	36 (39.1%)	–	–	–
Occurrence of UTI at 1 year^a^	48 (47.5%)	22 (22%)	–	$<0.0001$

^a^Calculated after censoring for death at 1 year.

**Table 2:** Organ function, key clinical parameters and immunosuppressive therapy in patients with urinary sepsis and urinary tract infection.

	Urosepsis (n = 101)	UTI (n = 100)	p-value
**Clinical data**			
Diagnosis of AKI	76 (75.2%)	41 (41%)	$<0.0001$
AKI stage 1	39 (38.6%)	35 (35%)	0.6615
AKI stage 2	16 (15.8%)	4 (4.0%)	0.0102
AKI stage 3	21 (20.8%)	2 (2.0%)	$<0.0001$
Need of RRT	17 (16.8%)	1 (1.0%)	0.0002
Mean arterial pressure (MAP) [mmHg]	93.3 (87–100)	96.7 (90–104)	0.0089
MAP $<70$ mmHg	10 (9.9%)	0 (0%)	0.0037
Vasopressor use	6 (5.9%)	0 (0%)	0.0394
Acute thrombocytopenia	32 (31.7%)	20 (20%)	0.0761
PLT $<150$ tys/ul	20 (19.8%)	16 (16%)	0.5817
PLT $<100$ tys/ul	12 (11.9%)	4 (4.0%)	0.0653
Consciousness by Glasow Coma Scale	14 (13–14)	15 (15–15)	$<0.0001$
Acute liver injury	10 (9.9%)	0 (0%)	0.0015
Multi-drug resistant strain infection	29 (28.7%)	19 (19%)	0.1363
Pulmonary congestion on admission	21 (20.8%)	9 (9%)	0.0283
Peripheral edema on admission	21 (20.8%)	19 (19%)	0.8600
Urinary obstruction^a^	24 (23.8%)	21 (21%)	0.7355
Need for DJ catheter implantation (or exchange)	8 (7.9%)	5 (5%)	0.2904
Need for red blood cell transfusion	15 (14.9%)	3 (3.0%)	0.0070
SOFA score on admission	4 (3–5)	1 (0–1)	$<0.0001$
Maximal SOFA score	4 (3–6)	1 (0–2)	$<0.0001$
ICU admission	5 (5.0%)	0 (0%)	0.0718
**Immunosupressive therapy**			
Tripple maintenance therapy prior to admission	78 (77.2%)	84 (84%)	
Treatment with tacrolimus/cyclosporine	68/33	66/34	0.9363
GKS bolus in acute phase	75 (74.2%)	36 (36%)	$<0.0001$
Immunosuppression reduction in acute phase	101 (100%)	41 (41%)	$<0.0001$
Reduction to steroid only	32 (31.7%)	2 (2%	$<0.0001$
Reduction from tripple to dual maintenance therapy	29 (28.7%)	17 (17%)	0.0349
Drug dose reduction only	40 (39.6%)	22 (20%)	0.0052

^a^Defined as one of the following: graft hydronephrosis, residual urine volume after urination $<50\;{\text {ml}}$ or urinary retention confirmed on ultrasound.

### Risk factors for AKI development

To determine risk factors for the occurrence of UTI-AKI during hospitalization, a multivariable logistic regression model was implemented—Table 3. Four independent predictors were identified: admission for urinary sepsis (aOR = 3.29; 95% CI 1.54–7.04; p = 0.002), serum albumin concentration below 3.3 g/dL (aOR = 2.42; 95% CI 1.20–4.88; p=0,013), suboptimal allograft function (for eGFR $<30$ ml/min/1.73 m$^{2}$-aOR = 7.07; 95% CI 1.60–31.2; p = 0.010) and urinary obstruction (OR = 2.76; 95% CI 1.09–6.98; p = 0.032).

**Table 3:** Multivariable logistic regression model with retrograde variable elimination for risk factors of AKI development in KTRs with UTI and urosepsis.

	Univariable p-value	Multivariable p-value	Adjusted odds-ratio (OR, 95% CI)
Admission for urinary sepsis	$<0.0001$	0.002	3.29 (1.54–7.04)
Serum albumin concentration $<3.3$ [g/dL]	0.0001	0.013	2.42 (1.203–4.878)
eGFR $>60$ ml/min/1.73 m$^{2}$	–	–	Reference
eGFR 30–60 ml/min/1.73 m$^{2}$	0.0019	0.413	0.692 (0.287–1.672)
eGFR $<30$ ml/min/1.73 m$^{2}$	0.0009	0.010	7.07 (1.604–31.15)
Urine outflow obstruction [level: YES]	0.0217	0.032	2.76 (1.090–6.976)
Length of stay [days]	0.0002	–	–
Pulmonary congestion on admission	0.0015	–	–
Serum hemoglobin concentration [g/dL]	0.0098	–	–
Heart failure	0.0140	–	–
Peripheral edema on admission	0.0865	–	–
History of recurrent UTI	0.2697	–	–
Donor [living/deceased]	0.3267	–	–
Induction treatment	0.3308	–	–
CIT [min]	0.5223	–	–
Sum of HLA mismatch	0.7399	–	–

### Recovery from AKI

**Figure 2:**
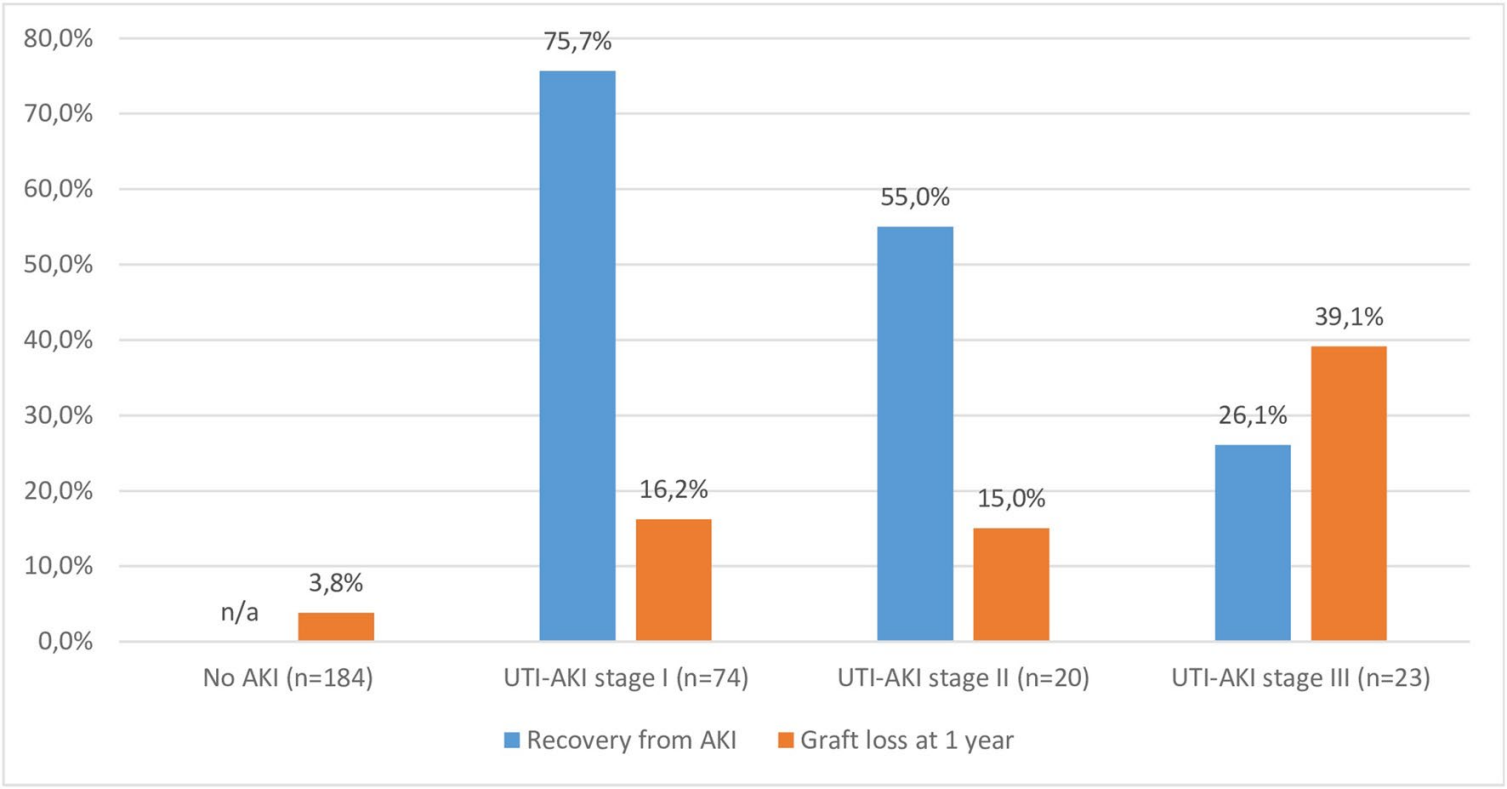
The incidence of recovery from AKI and graft loss according to the degree of acute kidney injury.

The rates of recovery from AKI stages 1, 2 and 3 equaled 75.6%, 55% and 26.1%, respectively—as presented in Fig. 2. Factors independently associated with renal recovery from AKI were AKI severity grade (AKI stage 2 with aOR = 0.25; 95% CI 0.078–0.792; p = 0.019 and AKI stage 3 with aOR = 0.1; 95% CI 0.026–0.335; $p<0.0001$), transfusion of PRBC (aOR = 0.22; 95% CI 0.055–0.904; p = 0.0360), and steroid bolus in the acute phase of treatment (aOR = 4; 95% CI 1.49–11.2; p = 0.006)—as presented in Table 4. The development of sepsis (US vs UTI group) did not influence the rates of renal recovery from AKI after adjustment for the remaining significant variables.

**Table 4:** Multivariable logistic regression analysis with retrograde variable elimination for predictors of recovery from UTI-AKI at 1-month post-discharge.

	Univariable p-value	Multivariable p-value	Adjusted odds-ratio (OR, 95% CI)
AKI stage 1	–	–	Reference
AKI stage 2	$<0.0001$	0.0190	0.248 (0.078–0.792)
AKI stage 3	$<0.0001$	$<0.0001$	0.094 (0.026–0.335)
Transfusion of blood products [level: YES]	0.0010	0.036	0.223 (0.055–0.904)
Time from KTx to entry [months]	0,0119	–	–
Reduction to steroid only [level: YES]	0.0127	–	–
Steroid bolus in acute phase [level: YES]	0.0150	0.006	4.09 (1.49–11.2)
Maximal SOFA score	0.0026	–	–
Pulmonary congestion on admission [level: YES]	0.0346	–	–
SOFA score on admission	0.0618	–	–
Mean baseline eGFR [ml/min/1.73 m$^{2}$]	0.5038	–	–
Urinary sepsis or UTI	0.8163	–	–

### Graft loss and graft function at 1 year

**Figure 3:**
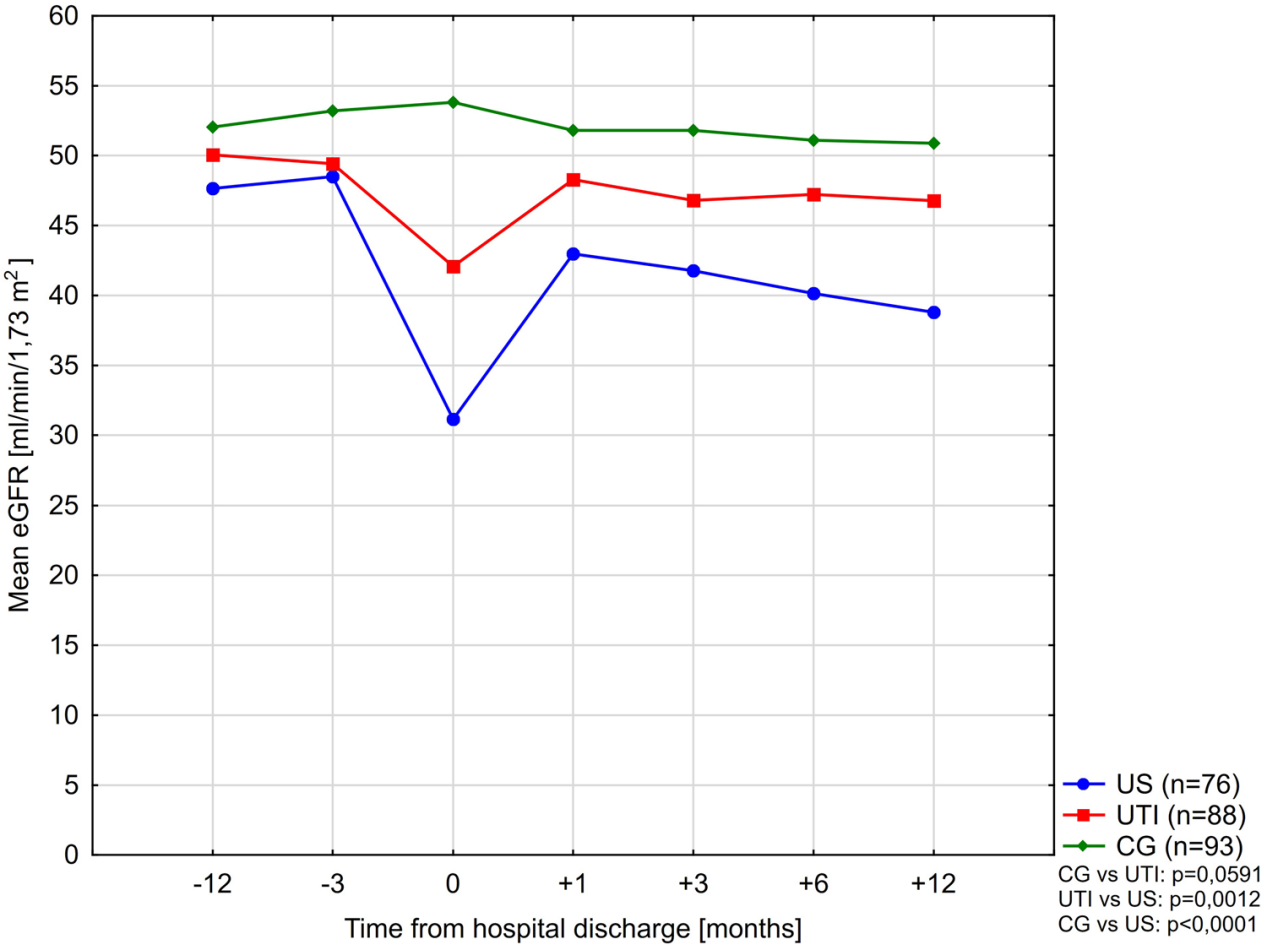
Mean eGFR values in the study groups during the observation period. Patients with graft loss registered at any point of the study were not included on the graph.

A complex comparison of eGFR values for the study groups, after excluding cases where graft loss was registered is presented in Table 5. In the control group, no significant decline of eGFR was observed after 1 years of observation. On the contrary, after 12 months from discharge, in both urosepsis and UTI groups, the estimated eGFR was significantly lower, with the urosepsis group showing a greater magnitude of decline. This observation has been confiremed by means of a conducted repeated measures ANOVA approach (within groups: df = 2, F = 8.047, p = 0.0004). More in-depth information on this analysis is provided in Section S3 of the Supplement. The eGFR trends are presented in Fig. 3. In multivariable analyzes, the admission for urinary sepsis and the diagnosis of AKI were also independently associated with a decline in eGFR at 1 year post-hospital discharge by $-2.55$ and $-5.28$ ml/min/1.73 m$^{2}$, respectively (Table 6). When distinguishing for AKI severity, AKI stage 1 did not show a significant impact on graft function, while AKI stages 2 and 3 were independently associated with increasing magnitude of eGFR impairment ($-8.2$ and $-9.2$ ml/min/1.73 m$^{2}$, respectively—Table 6).

**Table 5:** Calculated eGFR values (MDRD-4) in the study groups over time.

eGFR values over time	Urosepsis (n = 76)	UTI (n = 88)	CG (n = 93)	p-value
eGFR $-12$ months	47.6 15.61	50.1 14.25	52.0 13.53	0.1994
eGFR $-3$ months	48.5 15.27	49.4 13.49	53.2 13.42	0.0778
eGFR $+1$ months	42.9 14.29	48.3 16.57	52.9 15.01	$<0.0001$
eGFR $+3$ months	41.8 14.81	46.8 14.91	51.8 14.02	$<0.0001$
eGFR $+6$ months	40.1 14.3	47.2 15.26	51.1 13.73	$<0.0001$
eGFR $+12$ months	37.1 16.54	44.8 17.09	50.0 14.84	$<0.0001$
the p-value for first and last values comparison	$<0.0001$	0.0016	0.5723

**Table 6:** Multivariable Proportionate Cox Hazard model for predictors of death-censored graft loss at 1 year. Adjusted for time from transplantation and occurence of UTI. Adjusted R2 = 0.8925; 95% CI 0.8706–0.9164; $p<0.0001$.

	Univariable p-value	Unadjusted HR	Multivariable p-value	Adjusted HR (95% CI)
Time from KTx to entry [months]	0.0010	1.007	0.0651	–
AKI in the course of UTI or US [level: YES]	$<0.0001$	6.49	0.0140	6.31 (1.45–27.5)
FSGS as primary renal disease [level: YES]	$<0.0001$	7.67	0.0016	6.23 (1.99–19.44)
Reduction of immunosupression to steroid only [level: YES]	$<0.0001$	9.17	$<0.0001$	7.82 (3.18–19.25)
Transfusion of blood products [level: YES]	$<0.0001$	9.40	0.0005	5.76 (2.16–15.3)
Occurrence of UTI [level: YES]	0.0340	2.82	0.0977	–

**Figure 4:**
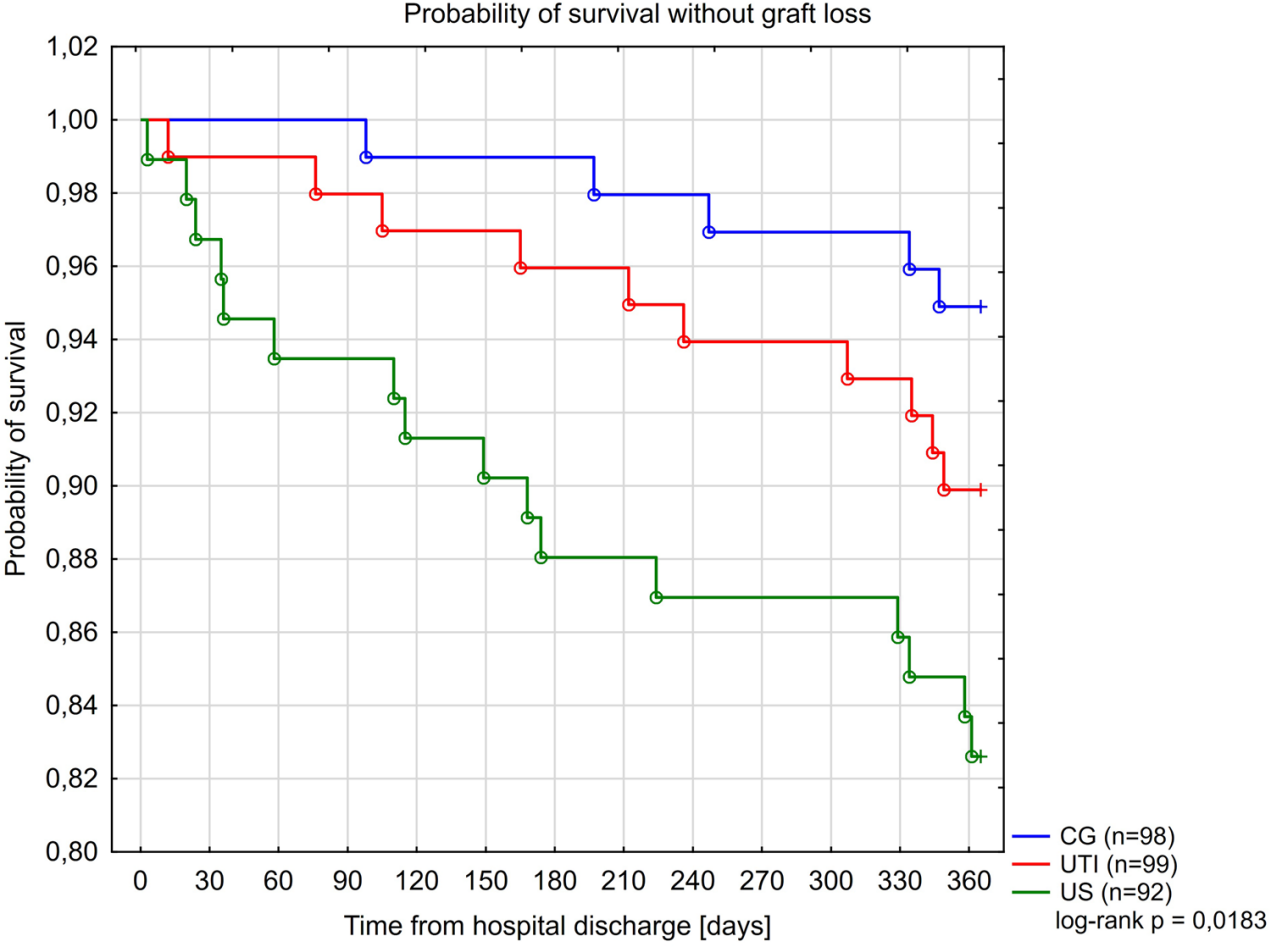
Kaplan Meier curves for survival without graft loss in the study groups.

**Figure 5:**
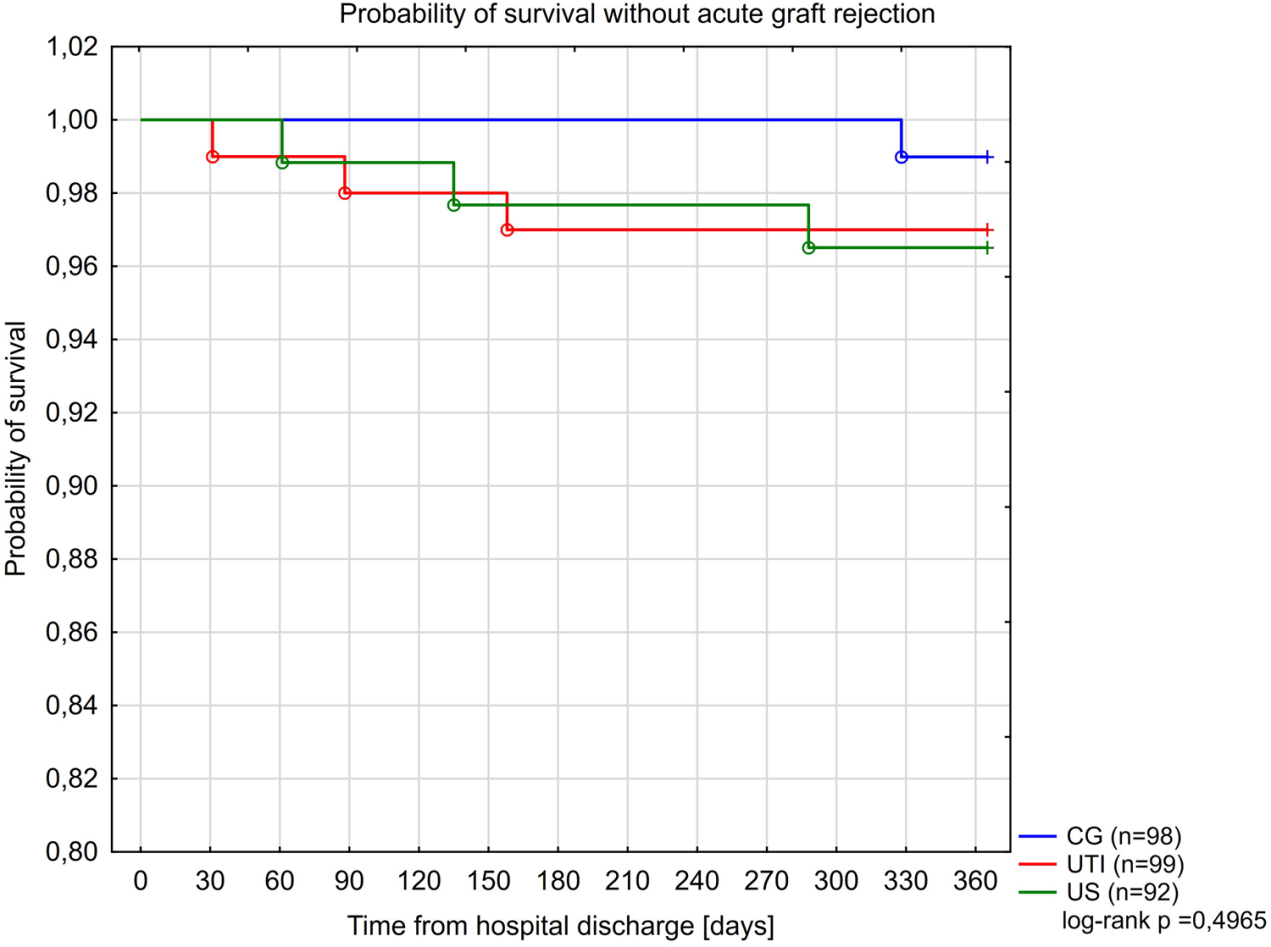
Kaplan Meier curves for survival without acute graft rejection in the study groups.

**Figure 6:**
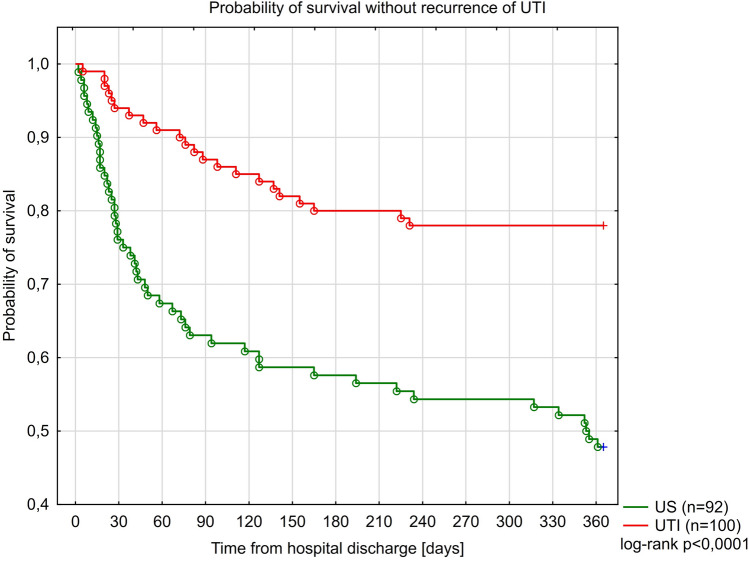
Kaplan Meier curves for survival without recurrence of UTI in the study groups.

Kaplan-Meier curves for the study groups for survival without death-censored graft loss (DCGL), acute rejection and recurrence of UTI, are presented in Figs. 4, 5 and 6. Only in 15 of 31 cases of graft loss we could reliably and specifically identify the underlying cause: UTI or urosepsis (n = 4) chronic graft hydronephrosis (n = 3), acute rejection (n = 3), recurrent kidney disease (n = 3), AKI (n = 1), chronic graft rejection (n = 1) and unknown cause (n = 16). Furthermore, four independent predictors of the occurrence of DCGL were identified: the reduction of immunosuppression in the acute phase of illness to steroid therapy alone (aHR = 7.82; 95% CI 3.18–19.3; $p<0.0001$), development of AKI (aHR = 6.31; 95% CI 1.45–27.5; p = 0.014), FSGS as a primary renal disease (aHR = 6.23; 95% CI 1.99–19.44; p = 0.0016) and transfusion of PRBC (aHR = 5.76; 95% CI 2.16–15.3; p = 0.0005)— Table 7. A total of 20 biopsies was performed in patients with failing grafts. A table presenting the frequency of graft loss causes in each of the study groups was presented in Section S2 (Table S1) in the Supplement to this article.

**Table 7:** Multivariable linear regression model for predictors of eGFR at 1-year post-discharge. Adjusted for the time from transplantation, amount of HLA mismatches and tripple mainenance therapy. Adjusted R2 = 0.6478; 95% CI 0.5803–0.7153; p$<0.0001$.

	Univariable p-value	Multivariable p-value	Regression coefficient	95% CI
Baseline eGFR [ml/min/1.73 m$^{2}$]	$<0.0001$	$<0.0001$	0.797	0.704–0.896
Time from transplantation [months]	0.0120	0.1259	–	–
Sum of HLA-AB i-DR mismatch	0.0308	0.7327	–	–
Tripple maintenance therapy on admission	0.0130	0.4588	–	–
Admission for urinary sepsis [level: YES]	$<0.0001$	0.0116	$-2.55$	($-0.52$)–($-4.10$)
Development of AKI [level: YES]	$<0.0001$	$<0.0001$	$-5.28$	($-2.90$)–($-6.36$)
AKI stage 1:	0.0012	0.3609	–	–
AKI stage 2:	$<0.0001$	0.0007	$-8.20$	($-3.47$)–($-12.9$)
AKI stage 3:	$<0.0001$	$<0.0001$	$-9.21$	($-6.01$)–($-12.4$)

## Discussion

Sepsis, including urosepsis, in transplant recipients seems to be associated with lower mortality than in the non-transplant population^24,25^. It is however a significant source of complications, as it may lead to allograft injury and its sequelae. To our best knowledge, this is one of the very few studies which analyze risk factors for the development of UTI-AKI in KTRs and its impact on long-term transplantation outcomes. It also adresses the impact of implemented treatment strategies, including the changes of immunosuppressive regimen in the acute phase of illness. The development of UTI-AKI was observed in 75% and 41% of patients in the urosepsis and UTI groups in our study, respectively. The distribution of each severity grade of AKI was different as well across these two groups, with a preponderance of AKI stages 2 and 3 in the urosepsis group. Multivariable analyses have shown that the occurrence of UTI-AKI was independently associated with both increased rates of graft loss at 1 year and a decline of eGFR at 1 year, independently of urinary tract infection occurrence. In patients with UTI who did not develop AKI, the recorded graft loss rates equaled the values registered in the general KTR population (of about 3–5% yearly)^26^. Therefore it may be possible that AKI, but not UTI alone, directly promotes renal graft failure and loss. According to the authors’ knowledge, reports on these two events studied alongside each other have not been published. It is also noteworthy that in the presented study, only AKI stages 2 and 3, but not AKI stage 1 (irrespectively of sepsis development) contributed to the impairment of allograft function, which remains in agreement with the observed high probability of renal function recovery in KTRs with AKI stage 1. Additionally, the recovery rates from AKI stages 2 and 3 were significantly lower than from AKI stage 1. Therefore, a hypothesis is proposed that AKI stage 1 related to UTI or urinary sepsis shows somehow benign character and may not influence the long-term outcome after infection, although such impact might have been revealed in an analysis of a larger patient cohort.

In our study, except for the development of AKI, three other independent predictors of DCGL have been identified: FSGS as primary kidney disease, the need for PRBC transfusion, and the reduction of immunosuppression in the acute phase of illness to standalone steroid therapy. The exposure to transfusion of red blood cells was also a negative predictor of renal recovery after UTI-AKI. Except for the recurrence of FSGS, these factors have not yet been described as potentially modifiable risk factors of kidney graft loss in the infection setting. According to the scarce literature, urinary sepsis and urinary tract infections contribute to 35% of AKI cases in KTRs^4^. Therefore, other alternative causes of AKI must always be sought proactively, as is is not uncommon for the AKI etiology to be multifactorial. Additional AKI causes in KTRs include: other severe infections (pneumonia, intraabdominal sepsis), intravascular volume depletion, viral infections (CMV and BKV), immune-mediated injury, calcineurin inhibitor toxicity (CNI), recurrence of primary kidney disease (FSGS and HUS), drug-induced microangiopathy, use of contrast media and other nephrotoxic drugs, graft vessel thrombosis, ureteral stenosis and surgical complications^27–30^. Furthermore, there is also a wide selection of risk factors portending occurrence of AKI in this patient group, which in turn include: suboptimal graft function, the need for mechanical ventilation, vasopressor use, the time of dialysis before transplantation, a history of delayed graft function and proteinuria^30,31^.

**Figure 7:**
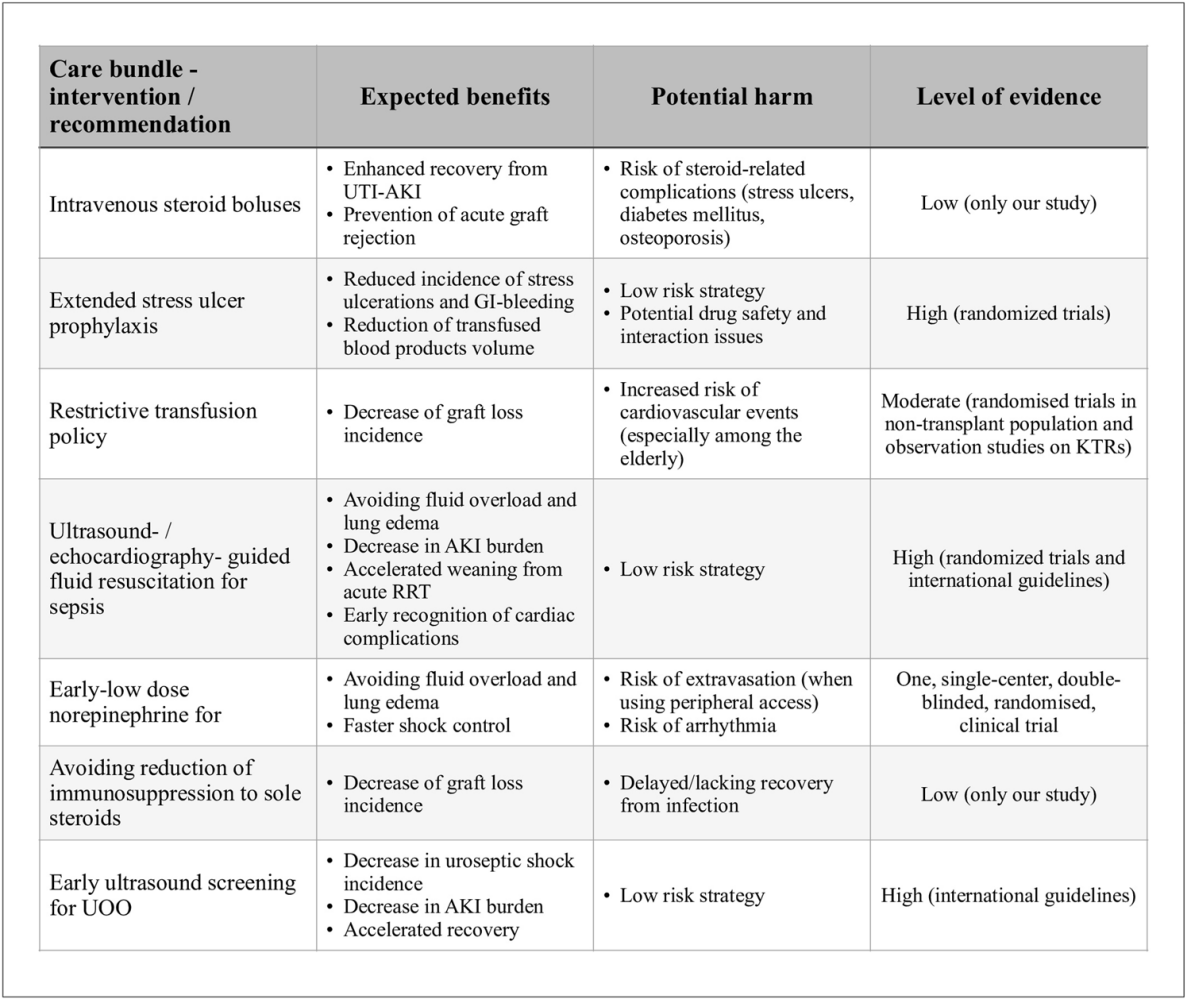
Summary of recommendations regarding acute care of KTRs with UTI and urosepsis.

Based upon the findings of this study and the performed literature review, several suggestions regarding the optimal management of acute UTI and urosepsis in KTRs outcomes are put forward. Each of these suggestions is discussed in the following sections and summarized (along with their strength and limitations) in a form of care bundle presented on Fig. 7.

First, as in 95% of the cases, the transfusion of PRBC concentrate was dictated by upper- or lower gastrointestinal bleeding with subsequent anemia, the authors of this study highlight the need for effective stress ulcer prevention in KTRs hospitalized with severe infections and sepsis. Despite concerns being constantly raised about the safety of early and extended stress ulcer prophylaxis (both with PPI and H2-blockers), we have failed to identify any study, where any damage resulting from such interventions has been shown^32^. Furthermore, the association between transfusions of blood products and impaired renal recovery as well as graft loss has also been clearly documented by the present authors. This observation confirms previous findings described in two recent studies, where peri-transplant PRBC transfusion was also associated with increased graft loss after 1 year^33,34^. Therefore, a restrictive transfusion policy in these cases may potentially improve recovery post-AKI by preventing alloimmunization and autoimmune response against kidney allograft which may occur after transfusion of blood products^35^. Furthermore, in the setting of septic shock, a liberal transfusion trigger does not seem to offer any advantages over a restrictive strategy, both in terms of 90-day survival and the rate of ischemic events^36,37^.

Secondly, the authors strongly advocate for the use of a stress dose of corticosteroids in KTRs admitted acutely to the hospital due to UTI or urosepsis (not only as prophylaxis of adrenal gland insufficiency but also as a nephroprotective therapy). In this study, the use of intravenous steroid boluses in the acute phase of illness was associated with increased odds of renal recovery after UTI-AKI, independeetly from stage of AKI. The rationale for such a strategy includes the fact that the stress dose of steroids in an acute infective or septic setting, may help optimize peripheral perfusion and prevent hypovolemia related to adrenal insufficiency in steroid-exposed patients, thus potentially preventing the development or aggravation of AKI^38,39^. Moreover, the use of additional steroids in KTRs may prevent the development and potentially treat the early stages of beginning acute graft rejection, minimizing immune-mediated injury to the kidney graft^40^. The standard dosing administered in our center was methylprednisolone every 12 h, dosed 20–40 mg daily until the improvement of clinical state was noted (median time of use equaled 3 days).

Thirdly, the reduction of immunosuppression in the acute phase of UTI and urosepsis to isolated steroid therapy seems to be associated with more frequent graft loss at 1 year. So far, the scientific literature does not support the hypothesis that any reduction of immunosuppression in KTRs with acute infection does enhance patients’ recovery. The authors of this study have identified no clinical trials addressing this topic and one propensity-matched retrospective study by Yahav et al., in which no clinical benefit, such as reduction of mortality, readmission rates, or enhanced recovery, has been observed^41^. On the contrary, many trials and observational studies have shown a clear association between the reduction of immunosuppression (both drug discontinuation and dose reduction) in the non-infective setting and increased incidence of acute rejections^42^. This observation remains in agreement with both the presented study and the study by Yahav et al. who have observed a trend towards increased graft loss rates in KTRs with acute infection in whom immunosuppression was discontinued (however they do not state the extent of drug discontinuation)^39^. As the study presented in this paper probably underestimates the frequency of AGR occurrence, no association between the reduction of immunosuppression and AGR has been observed (out of 31 cases of graft loss, in 15 graft biopsies AGR was reported in 3 cases). Several studies have already shown that acute graft rejection may have an asymptomatic-subacute course and may remain undiagnosed in KTRs until symptomatic graft failure is developed^43^. Therefore, based upon the observations of this study and available literature, the routine reduction of immunosuppression to sole steroid regimen in the acute phase of infection is not recommended as it may portend detrimental transplant outcomes.

Fourthly, about 20% of KTRs with urinary sepsis after initial fluid resuscitation in our study, have presented with clinical signs of pulmonary and peripheral congestion on admission to the Transplantation Unit. Meanwhile, the rates of lacking shock control and vasopressor requirement after fluid resuscitation were very low as compared to types of sepsis other than urosepsis (where the frequency of these complications reaches 30–50%)^2^. There is also increasing evidence that a positive fluid balance in the course of sepsis, AKI and other acute diseases is a strong and independent predictor of poor clinical outcomes^44–47^. Kidney transplant recipients are in turn particularly susceptible to excessive fluid accumulation due to a decreased filtration reserve, frequent UOO, and cardiovascular disease (either heart failure and coronary or peripheral arterial disease). Furthermore, the evidence documenting damage inflicted by volume-underdosing is scarce, except the setting of major elective abdominal surgery, where restrictive fluid strategy is associated with increased incidence of AKI^48^. In a recently published single-center randomized controlled trial by Permpikul et al., the authors have randomized 310 adult patients with sepsis and hypotension in the emergency department to either standard fluid resuscitation versus standard fluid regimen and low dose norepinephrine infusion^49^. In the norepinephrine group, the shock control rate at 6 h was significantly higher than in the control group, but also the incidence of new-onset arrhythmia and cardiogenic pulmonary edema was significantly lower (with an equal amount of administered crystalloid fluids). It is, therefore, possible that early low-dose norepinephrine administration in septic-hypotensive patients may allow to reduce the number of complications related to fluid overload (especially in KTRs who show increased susceptibility to water retention) and achieve quicker shock control—by keeping the administered fluid volume intravassaly. This in turn may prevent the development of septic hypovolemia and provide better perfusion of the renal allograft (which lacks autonomic innervation and autoregulation mechanisms)—thus preventing the development of progression of UTI-AKI.

In secondary analyses, several risk factors associated with the development of UTI-AKI have also been identified. These included: admission for urinary sepsis, hypoalbuminemia, suboptimal allograft function, and urine outflow obstruction (UOO) which are well-known and documented factors that increase the odds of developing acute kidney injury^2,16,31,50^. Studies show that even 75% of cases of urinary sepsis in the general population are induced by urine outflow obstruction, which might as well be the case in KTRs^51^. According to previous studies, the early recognition and relief of urine obstruction may allow for reduction of AKI-related burden and even prevent the development of urosepsis and uroseptic shock^52^. The relief of UOO in case of urinary sepsis should thus be treated as “source control” which, alongside fluid resuscitation and administration of antimicrobials, remain the cornerstone of successful sepsis treatment^53,54^. Therefore it is strongly recommended that early ultrasonographic screening for urine outflow obstruction be conducted in every KTR admitted to the hospital for any UTI or acute infection.

Lastly, a potentially modifiable risk factor for AKI is hypoalbuminemia. Both pre- and post-transplant hypoalbuminemia remain an independent factor portending allograft loss as well. However, the substitution of albumins in kidney transplant recipients (irrespectively of whether considered in acute or non-acute setting) remains controversial. Studies show that hypoalbuminemia in KTRs is developed almost exclusively due to renal protein loss which occurs due to primary glomerular kidney pathology (predominantly FSGS and membranous nephropathy) or due to sirolimus treatment^55,56^. Therefore, in KTRs with significant urine output and significant albuminuria, the supplementation of albumin may frequently be ineffective in terms of normalizing albuminemia and for that reason should not be routinely implemented. However, such an intervention should be considered in KTRs with sepsis and lacking shock-control as an adjunct for fluid resuscitation as stated in SSC 2016 Guidelines^57–59^. This allows hypothesizing that the relationship between hypoalbuminemia and late allograft loss is partially due to preexisting glomerulopathy but also secondary to increased incidence of AKI or altered pharmacokinetics of immunosuppressive drugs^60^.

It is acknowledged that there are several limitations to this study. First of all, a non-random selection bias can not be excluded due to the retrospective-observational character of the study. Second, the relatively small sample size might have caused underpowering and underestimations of the impact of some variables on transplantation outcomes. What is more, eGFR values used for patient follow-up and diagnosis of AKI were based on serum creatinine measurements, which is an imperfect and labile marker of kidney filtration function. Although newer and more accurate markers as cystatine C or IGFBP-7 are known, their use remains limited only to clinical trails^61^. Furthermore, the results presented on Fig. 3 should be interpreted with caution, due to the fact that censoring patients who have lost their kidney graft might have caused under- or overestimation of plotted mean eGFR values. Finally, the above-mentioned remarks regarding the optimization of long-term treatment outcomes are merely hypothesis-generating suggestions, which cannot replace internal treatment protocols but are intended to indicate the course of future research. However, in absence of any international guidelines on acute care in KTRs, this is also the first study that focuses on optimal management of these patients in the acute setting. It is also stressed that to the knowledge of the authors, this is the first study to analyze the outcomes of UTI-rated acute kidney injury in kidney transplant recipients, allowing the identification of modifiable risk factors which have not yet been described in the literature in this setting. Despite certain limitations, this study presents unique and valuable observations which we believe, have the potential to decrease the renal burden and prevent such complications as allograft loss in KTRs in the future.

## Supplementary information

Supplementary Information.

## Data Availability

The dataset supporting the conclusions of this article is available in the Mendeley repository under 10.17632/m323c88sy4.2.
