# Supplementary material for: Acute kidney injury secondary to urinary tract infection in kidney transplant recipients
Source: Sci Rep. 2022 Jun 27;12:10858. doi: 10.1038/s41598-022-15035-7 (PMC9237017; doi:10.1038/s41598-022-15035-7)
Supplement: Supplementary file 1 — Supplementary Information. [file 41598_2022_15035_MOESM1_ESM.pdf]

S1 - Tukey plots and Bland Altman analyzis of eGFR calculated from MDRD-4 and CKD-EPI equations in the study dataset

Adequate Tukey plots obtained from Bland Altman analyzis are included on Figures S1-S6.

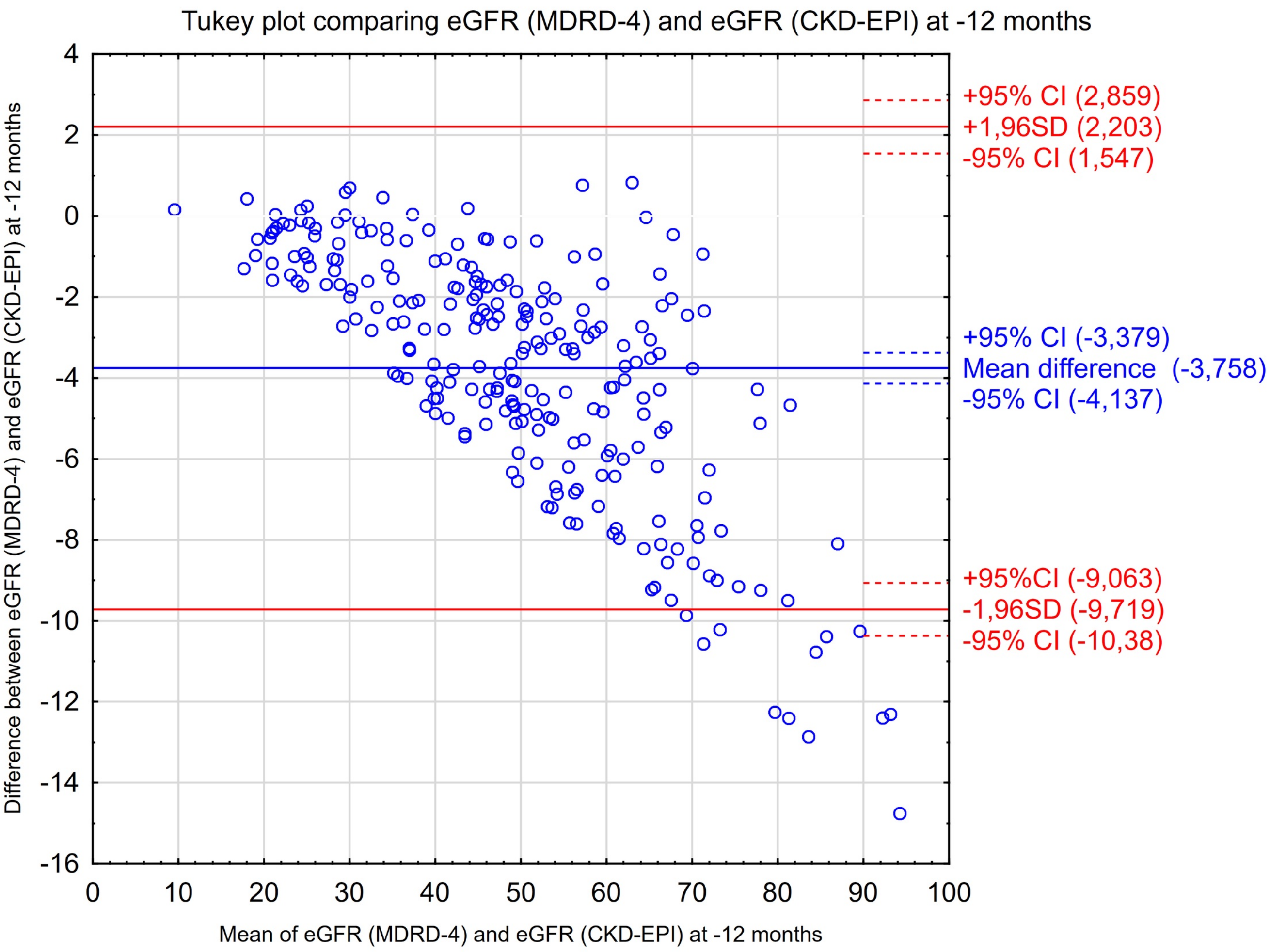

Figure S 1. Tukey plots comparing eGFR calculated in the whole study population with MDRD-4 and CKD-EPI equations at -12 months

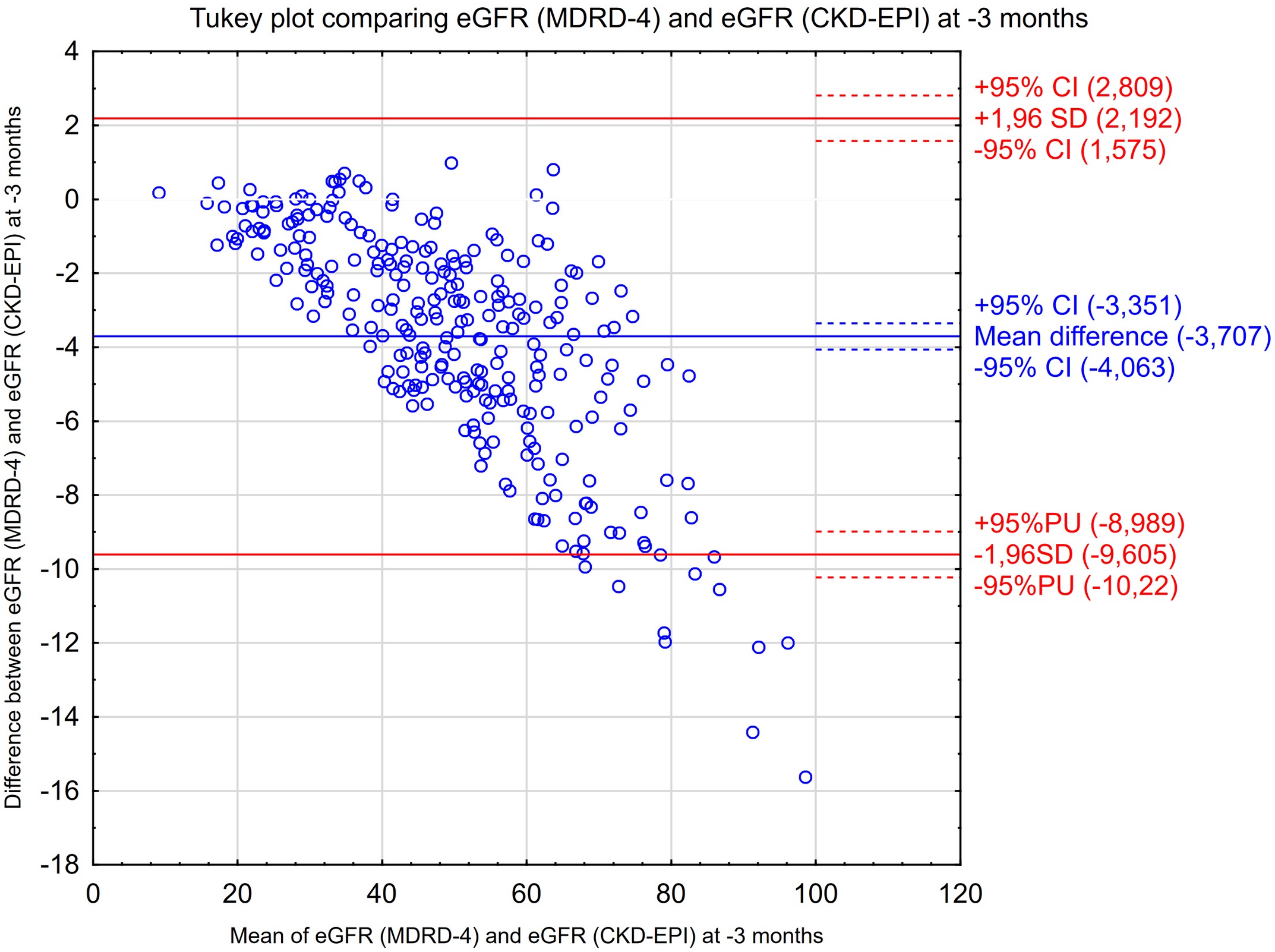

Figure S 2. Tukey plots comparing eGFR calculated in the whole study population with MDRD-4 and CKD-EPI equations at -3 months

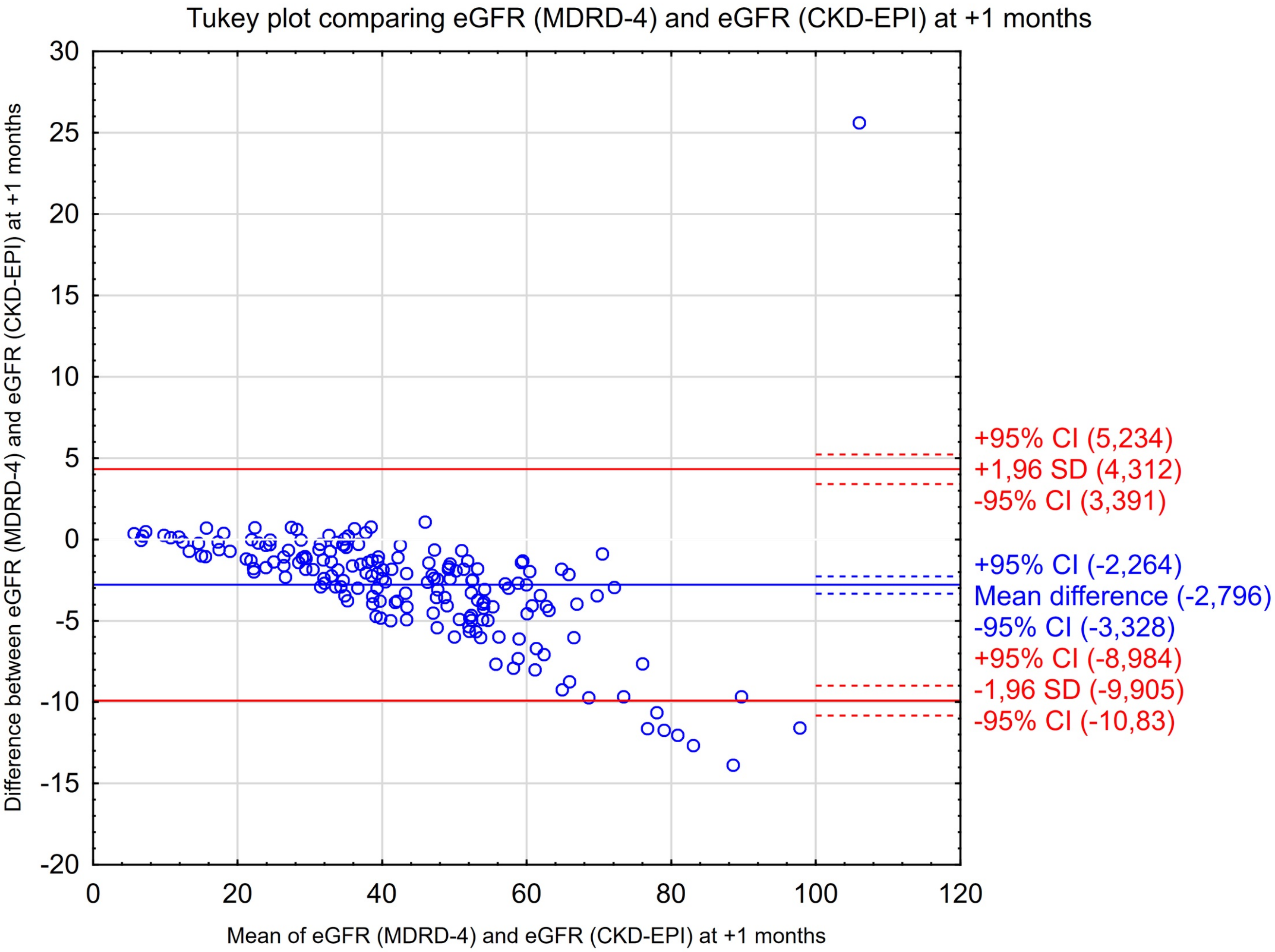

Figure S 3. Tukey plots comparing eGFR calculated in the whole study population with MDRD-4 and CKD-EPI equations at +1 months

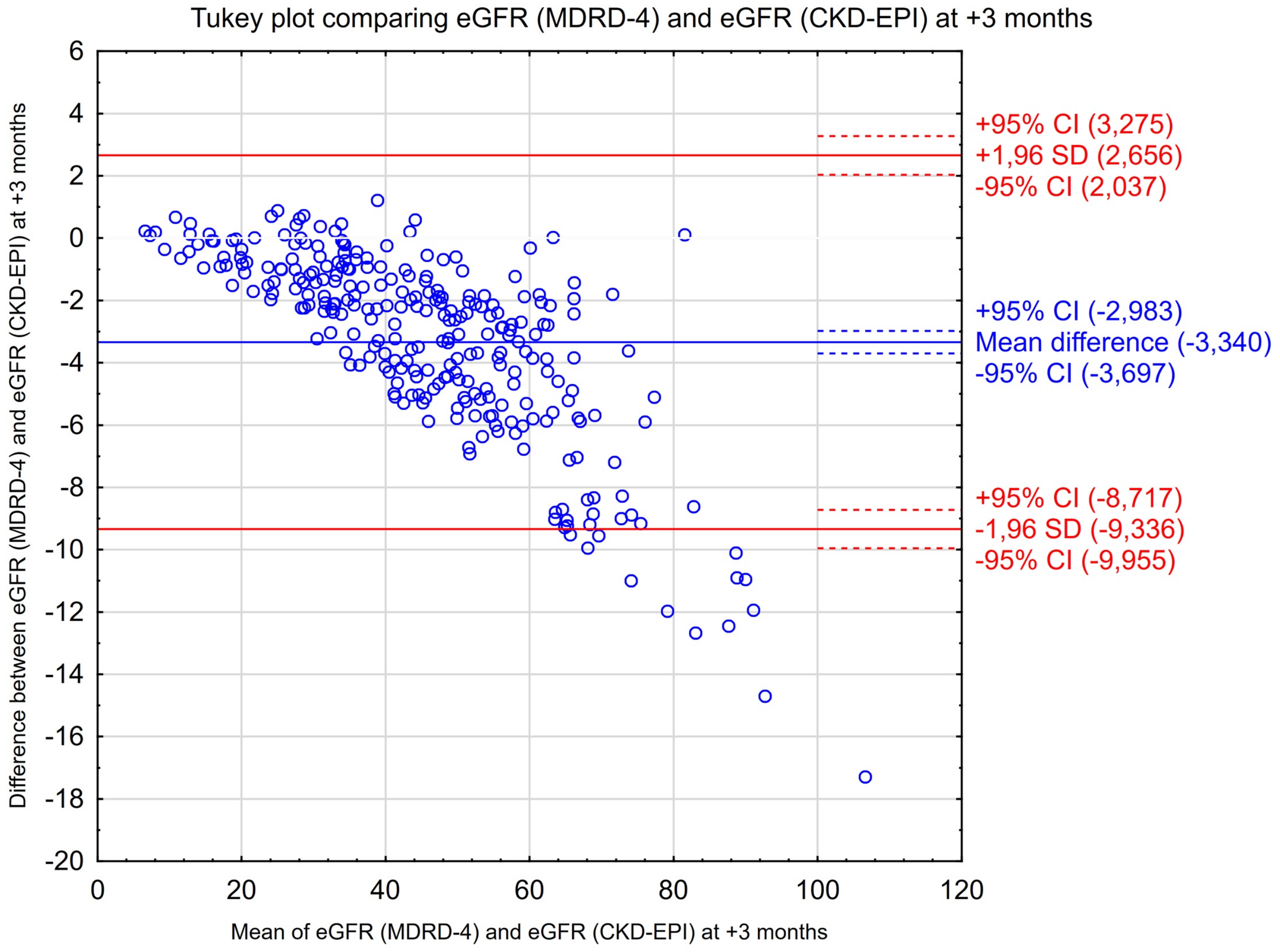

Figure S 4. Tukey plots comparing eGFR calculated in the whole study population with MDRD-4 and CKD-EPI equations at +3 months

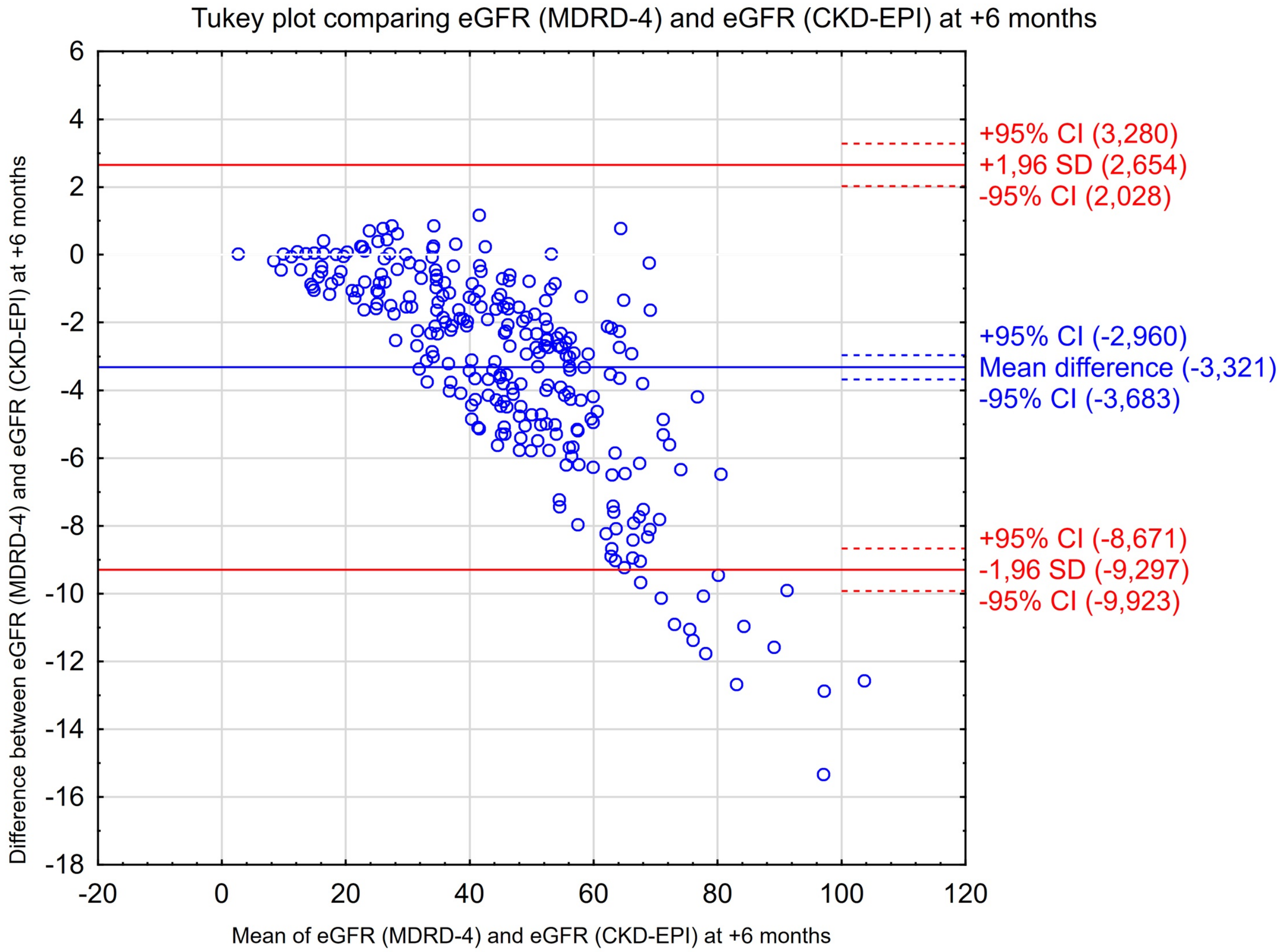

Figure S 5. Tukey plots comparing eGFR calculated in the whole study population with MDRD-4 and CKD-EPI equations at +6 months

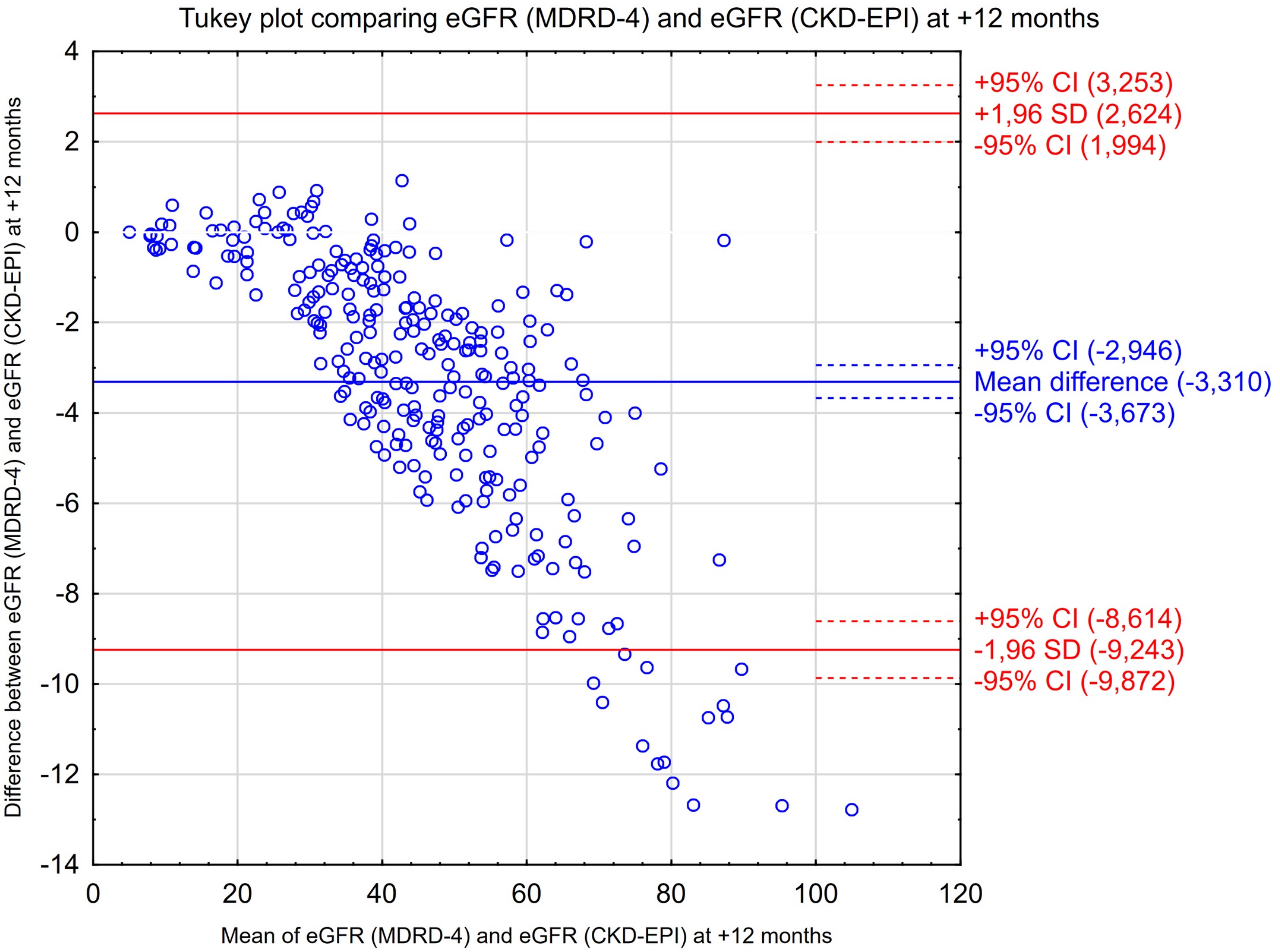

Figure S 6. Tukey plots comparing eGFR calculated in the whole study population with MDRD-4 and CKD-EPI equations at +12 months

S2 - The causes of graft loss across the study group

The causes of graft loss could not be specified in each patient in the database. The summary of graft loss cause in all study groups are presented in the table below. A total of 20 biopsies was performed in patients with failing grafts.

Causes of graft loss	US-group	UTI-group	Control group	Total
UTI (uncluding urosepsis)	3 (18.75%)	1 (10%)	0 (0%)	4 (12.9%)
Chronic graft hydronephrosis	1 (6.25%)	2 (20%)	0 (0%)	3 (9.7%)
Acute rejection	0 (0%)	2 (20%)	1 (20%)	3 (9.7%)
Recurrent kidney disease	2 (12.5%)	1 (10%)	0 (0%)	3 (9.7%)
Non-septic AKI	0 (0%)	1 (10%)	0 (0%)	1 (9.2%)
Chronic graft rejection	1 (6.25%)	0 (0%)	0 (0%)	1 (9.2%)
Unknown cause	9 (56.25%)	3 (30%)	4 (80%)	16 (51.6%)
Total	16 (100%)	10 (100%)	5 (100%)	31 (100%)

Table S 1. Causes of graft loss across all study groups.

S3 - Results of the repeated measures ANOVA for serial eGFR measurments in the study groups

Additionally to simple students t-test comparisons of eGFR values presented in Table 5, we have also performed a repeated measures ANOVA analyzis, which has confirmed that eGFR values over time have been changing statistically significant between the study groups. The Table below presents the obtained results. Following parameters were applied: independent variables - eGFR values over time, number of repeated measures - 6 times, dependent variable - US/UTI/Control group. The patients included in the analyzis were not censored for graft loss. Due to violated assumption of sphericity (Maulchy's W=0,1851, df=14, p<0,01) a Bonferroni correction was utilized. The models parameters are presented in Table S2. Figure S7 presents the comparison of pooled estimated marginal means of eGFR values between the study groups, meanwhile Figures S8-10 present the estimated marginal means of eGFR values over time in each of the study group separately.

	SS	df	MS	F	p-value
Between groups	2635730	1	2635730	2078.4	<0.0001
Within groups	20411	2	10205	8.047	0.0004
Error	261242	206	1268		
Total	35543	1030	35		

Table S 2. Repeated measures ANOVA analyzis for repeated eGFR measurments.

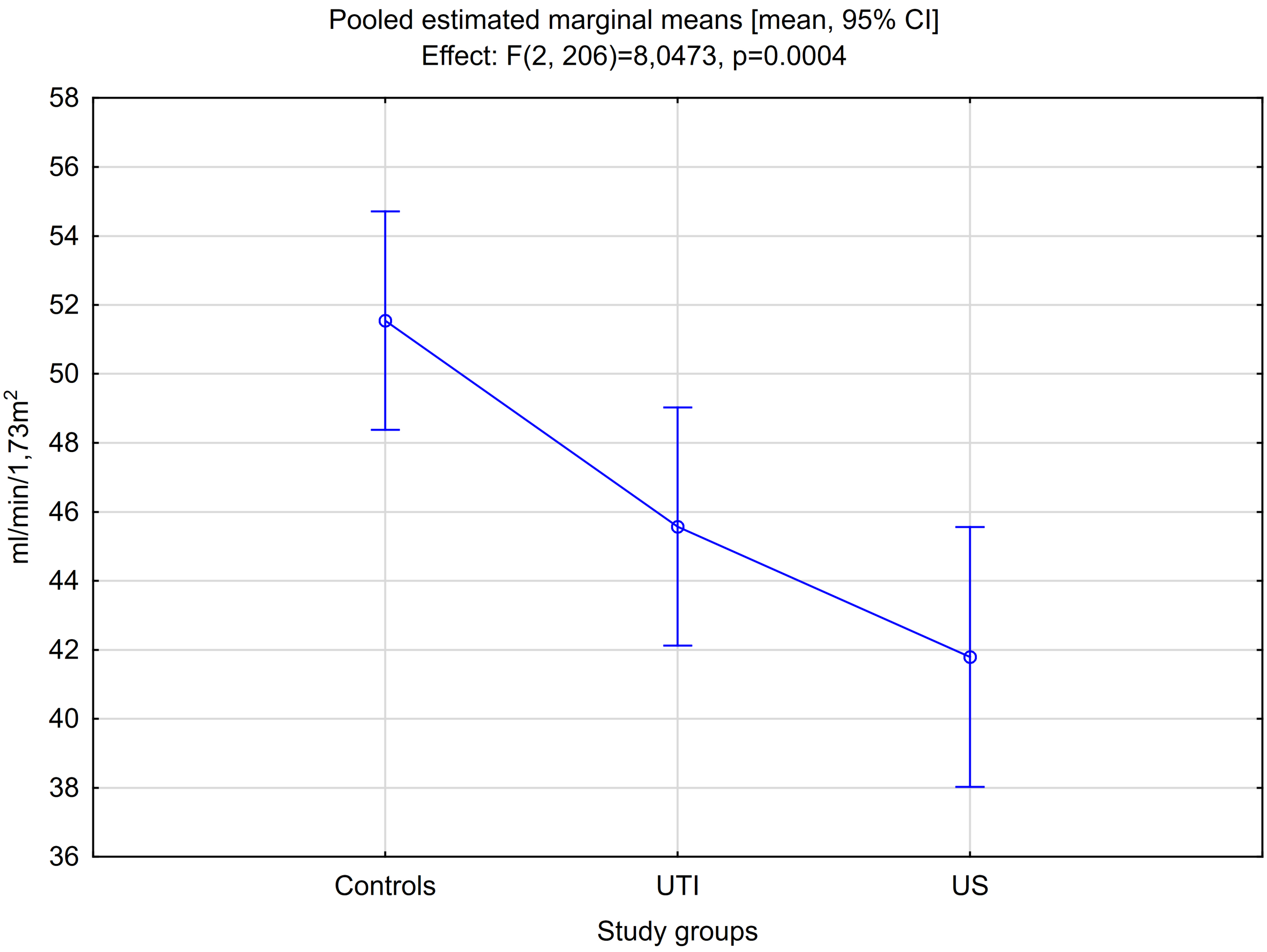

Figure S 7. Pooled estimated marginal means of the eGFR values in the study groups.

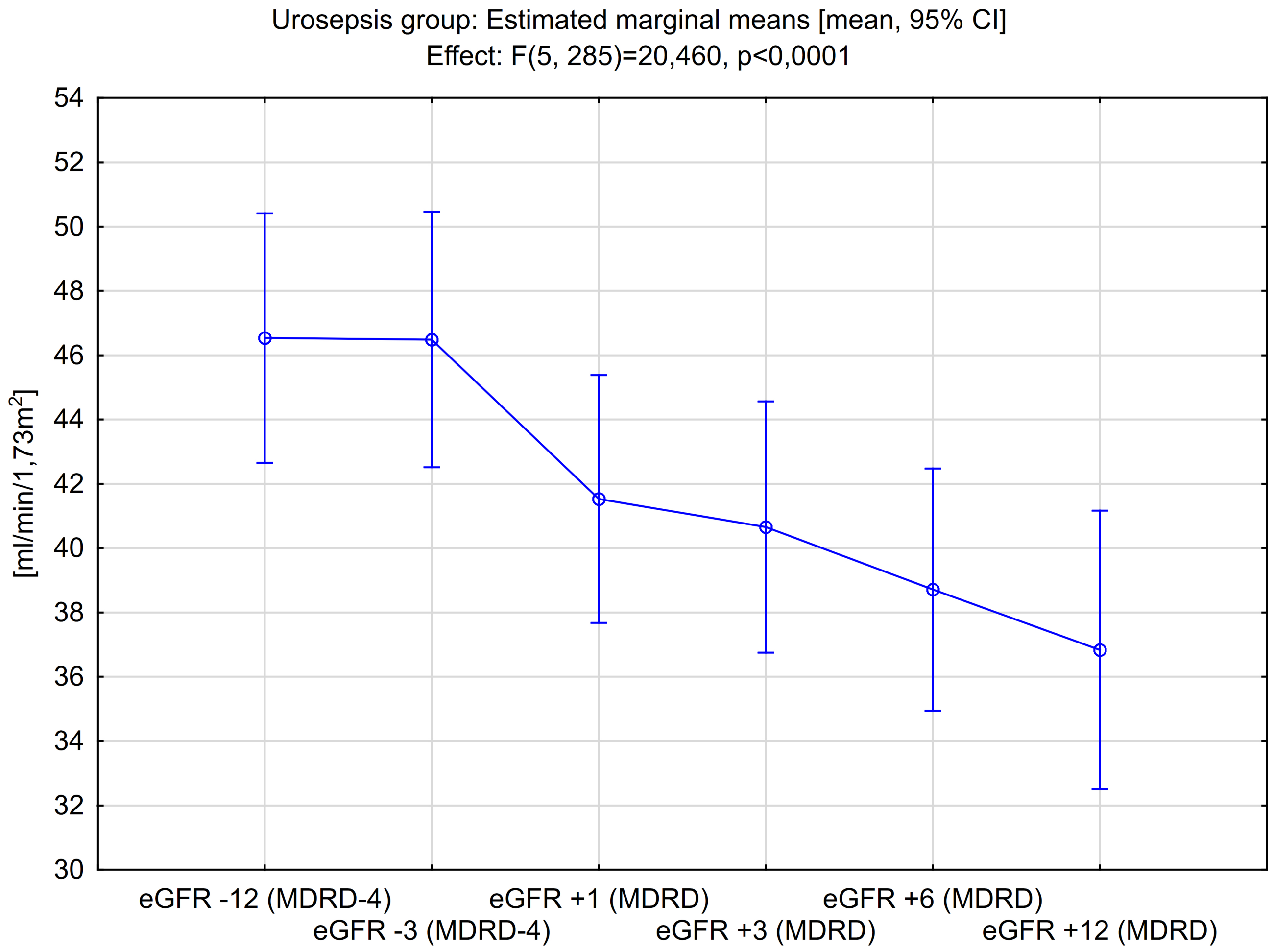

Figure S 8. Estimated marginal means of the eGFR values in the US group.

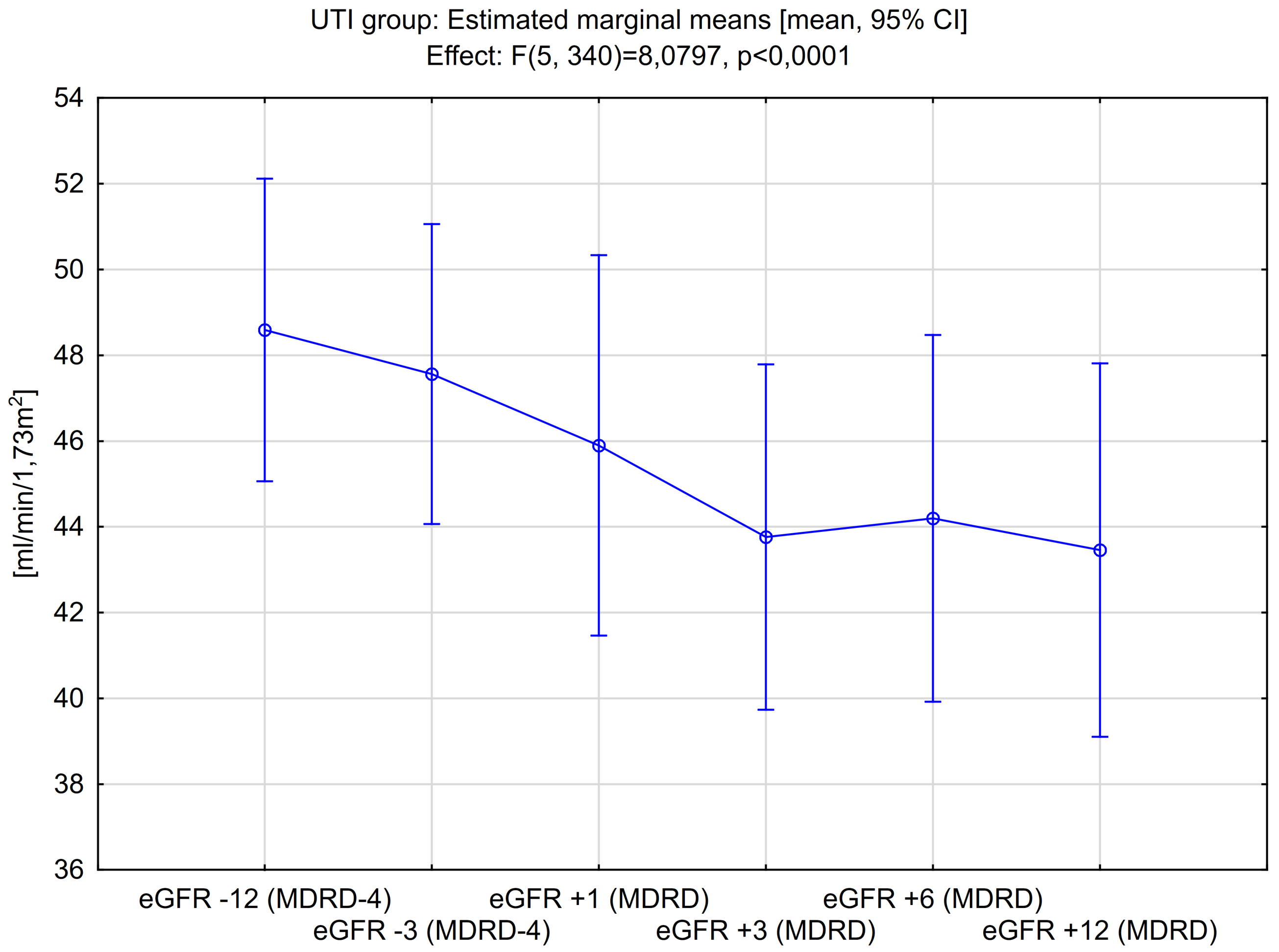

Figure S 9. Estimated marginal means of the eGFR values in the US group.

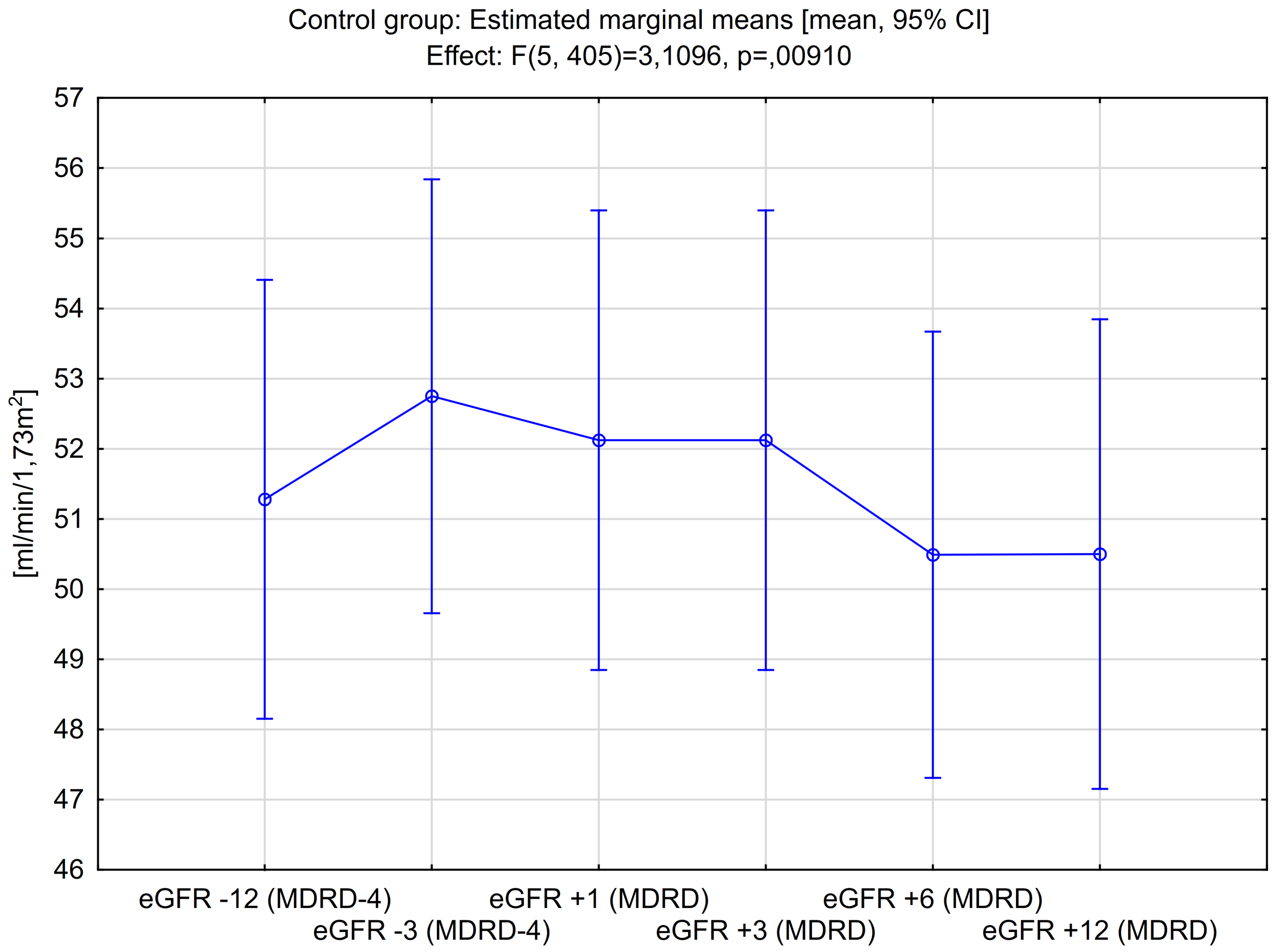

Figure S 10. Estimated marginal means of the eGFR values in the control group.
